# Effectiveness of Adapted COVID-19 Vaccines and Ability to Establish Herd Immunity against Omicron BA.1 and BA4-5 Variants of SARS-CoV-2

**DOI:** 10.3390/vaccines11121836

**Published:** 2023-12-10

**Authors:** Pedro Plans-Rubió

**Affiliations:** 1 Public Health Agency of Catalonia, Department of Health of Catalonia, 08005 Barcelona, Spain; pedro.plans@gencat.cat; 2 Ciber of Epidemiology and Public Health (CIBERESP), 28028 Madrid, Spain

**Keywords:** adapted COVID-19 vaccines, effectiveness of COVID-19 vaccines, herd immunity against SARS-CoV-2, SARS-CoV-2, Omicron BA.1, Omicron BA.4, Omicron BA.5, COVID-19 vaccination

## Abstract

The emergence of novel SARS-CoV-2 variants has raised concerns about the ability of COVID-19 vaccination programs to establish adequate herd immunity levels in the population. This study assessed the effectiveness of adapted vaccines in preventing SARS-CoV-2 infection and the ability of the adapted vaccines to establish herd immunity against emerging Omicron variants. A systematic literature review was conducted to estimate the absolute vaccine effectiveness (aVE) in preventing SARS-CoV-2 infection using adapted vaccines targeting Omicron variants. The ability of the adapted vaccines to establish herd immunity was assessed by taking into account the following factors: aVE, Ro values of SARS-CoV-2 and the use of non-pharmacological interventions (NPIs). This study found meta-analysis-based aVEs in preventing severe disease and SARS-CoV-2 infection of 56–60% and 36–39%, respectively. Adapted vaccines could not establish herd immunity against the Omicron BA.1 and BA.4-5 variants without using non-pharmacological interventions (NPIs). The adapted vaccines could establish herd immunity only by achieving >80% vaccination coverage, using NPIs with greater effectiveness and when 20–30% of individuals were already protected against SARS-CoV-2 in the population. New adapted COVID-19 vaccines with greater effectiveness in preventing SARS-CoV-2 infection must be developed to increase herd immunity levels against emerging SARS-CoV-2 variants in the population.

## 1. Introduction

The pandemic associated with the severe acute respiratory syndrome coronavirus 2 (SARS-CoV-2) is a worldwide public health challenge. Globally, as of 30 November 2023, 772 million laboratory confirmed cases of coronavirus disease 2019 (COVID-19) and 6.98 million deaths have been registered [1]. COVID-19 vaccination has been the key prevention strategy for the prevention and control of SARS-CoV-2 [2,3,4,5]. As of 23 November 2023, the percentages of COVID-19 vaccination coverage were 64.9% for full vaccination, 35.1% for booster doses and 70.6% for at least one dose of vaccine [6]. 

Based on COVID-19 trends, the World Health Organization declared on 5 May 2023 that COVID-19 was no longer a global health emergency [7]. The lifting of the Public Health Emergency of International Concern status by the WHO on 5 May 2023 signaled the start of a new phase of the global pandemic’s response and recovery, during which the WHO regional office for Europe shifted towards a longer-term strategy for SARS-CoV-2 prevention and control [8]. 

The two main objectives of COVID-19 vaccination programs are to protect vaccinated individuals and to establish and maintain herd immunity to block SARS-CoV-2 transmission in the community [2,3,5,9]. COVID-19 vaccination provides direct protection to vaccinated individuals by generating humoral and cellular immune responses and provides indirect herd immunity protection to unvaccinated and unprotected individuals [5,9]. The WHO considers that a vaccination coverage objective of 70% was adequate for achieving sufficient anti-SARS-CoV-2 herd immunity levels worldwide [3,10]. 

A study carried out in 2022 found that percentages of vaccination coverage in countries and regions of the WHO should be higher than 90%, and vaccination effectiveness in preventing infection should be higher than 88% to generate sufficient herd immunity to block SARS-CoV-2 with higher transmissibility [5]. The study suggested that, in the new socio-economic normality, countries should decide their COVID-19 vaccination coverage objectives based on taking into account the critical percentages of vaccination coverage that could prevent and block SARS-CoV-2 transmission [5]. 

COVID-19 vaccination programs using vaccines targeting the ancestral strain of SARS-CoV-2 reduced COVID-19-related morbidity and mortality [1,4,6]. Nevertheless, the emergence of novel SARS-CoV-2 variants has raised concerns about the ability of COVID-19 vaccines to block SARS-CoV-2 transmission in the population [11]. Firstly, since 2020, new pandemic waves caused by emerging SARS-CoV-2 variants (Alpha, Beta, Delta and Omicron) have occurred worldwide [1,12]. Secondly, the vaccine effectiveness against severe COVID-19 disease and SARS-CoV-2 infection has declined over time due to a combination of factors, including waning vaccine immunity and the immune evasion of emerging variants [13,14,15,16,17]. The COVID-19 vaccines available since 2020 were developed on the basis of the wild-type sequence of the SARS-CoV-2 spike protein, but emerging SARS-CoV-2 variants have acquired mutations reducing vaccine effectiveness and increasing viral transmissibility [13,14,15,16,17]. Since 2022, Omicron viruses account for over 98% of the publicly available sequences and it has diversified into several subvariants, including BA.5, BA.2 and XBB [12]. Thirdly, the immunity response after vaccination or infection is lower among individuals with chronic conditions, immune suppression and aged more than 65 years [15,18]. Fourthly, percentages of COVID-19 vaccination coverage achieved in children and infants are usually lower than those necessary to establish herd immunity and prevent SARS-CoV-2 transmission among children and infants [5,6,19]. 

The situation has changed with the development of adapted COVID-19 vaccines that target emerging variants, as they can achieve levels of vaccine effectiveness higher than those achieved with vaccines targeting the wild-type virus. On September 2022, bivalent COVID-19 mRNA vaccines, composed of components from the SARS-CoV-2 wild-type and Omicron BA.4/BA.5 (Pfizer-BioNTech vaccine) and BA.1 (Moderna vaccine) strains, were recommended by the EMA and the Advisory Committee on Immunization Practices (ACIP) of the United States to address the reduced effectiveness of COVID-19 monovalent vaccines during the SARS-CoV-2 Omicron variant predominance [20,21]. In September 2023, monovalent COVID-19 mRNA vaccines, composed of components from the SARS-CoV-2 Omicron XBB (Pfizer-BioNTech, Moderna) strains, were recommended [20,21]. The initial recommendation included persons aged 12 years or more (Pfizer-BioNTech) and 18 years or more (Moderna) who had completed at least a primary series of an approved monovalent vaccine (with or without subsequent booster doses). Several studies have found that booster vaccination with adapted vaccines targeting BA.4/BA.5 and BA.1 are associated with levels of vaccine effectiveness at preventing infection and severe disease ranging from 46% to 88% [22,23,24,25,26,27]. 

The impact of emerging variants on the population’s health depends on the population immunity and use of preventive measures. Given that present and future emergent variants could represent a serious threat to public health efforts to control SARS-CoV-2 epidemic waves, public health experts and policymakers have highlighted the need to develop adequate prevention strategies based on COVID-19 vaccination and non-pharmaceutical measures to reduce the socioeconomic and health impacts of new emerging variants worldwide [2,3,10]. Non-pharmaceutical measures, including mandatory face masks, social distancing and mobility restrictions, can be used to reduce SARS-CoV-2 transmissibility [28,29]. The objectives of this study were (1) to estimate the effectiveness of the adapted COVID-19 vaccines in preventing SARS-CoV-2 infection and (2) to assess the ability of adapted vaccines to establish herd immunity against the Omicron BA.1 and BA.4-5 variants of SARS-CoV-2.

## 2. Materials and Methods

### 2.1. Effectiveness of Adapted Vaccines in Preventing SARS-CoV-2 Infections

A systematic literature review of evidence on the absolute vaccine effectiveness at preventing SARS-CoV-2 infections and severe disease using adapted vaccines targeting Omicron variants was conducted following the PRISMA (preferred reporting items for systematic review and meta-analysis) guidelines [30]. The database search was performed on 4 and 5 October 2023 in PubMed using the terms “bivalent vaccine”, “vaccine BA.1”, “vaccine BA.4”, “vaccine BA.5”, “vaccine XBB”, “vaccine efficacy”, “vaccine effectiveness”, “absolute VE” and “VE”. The ROBIS (risk of bias in systematic review) tool was used to assess the risk of bias of the selected studies [31]. 

The inclusion criteria were as follows: (1) evaluative studies assessing the absolute effectiveness (aVE) of the adapted vaccines in preventing severe COVID-19 disease or SARS-CoV-2 infection; (2) studies published in English in peer-reviewed journals from October 2022 to 4 October 2023; and (3) studies that included individuals aged 18 years or more or 12 years or more. 

The effectiveness in terms of prevention of Omicron infection was defined as the vaccine’s ability to stop SARS-CoV-2 transmission from one person to another. When SARS-CoV-2 transmission is blocked, exposed unprotected individuals will not contract the virus and will not develop symptoms or disease. The effectiveness in preventing severe disease was defined as the ability of a vaccine to prevent hospitalization, critical care and serious symptoms among exposed individuals.

The absolute vaccine effectiveness (aVE) in preventing severe disease and SARS-CoV-2 infection is the proportion of severe disease and infection prevented in individuals vaccinated with the new vaccine regimen, including the adapted vaccines. It is measured by comparing health outcomes in vaccinated and in unvaccinated individuals (zero doses of vaccine). By contrast, the relative vaccine effectiveness (rVE) is the proportion of residual disease or infection remaining after the first regimen with vaccines targeting the wild-type virus that is prevented by the second vaccine regimen, including booster doses of adapted vaccines. 

Studies assessing the rVE were not selected to assess the aVE of the adapted vaccines because the aVE may not be comparable when the absolute VE varies for the comparator vaccine. The absolute reduction in preventing severe disease or infection using the adapted vaccines compared with the older one based on vaccines targeting the wild-type virus can be different in studies reporting the same rVE, depending on the aVE of the older regimen. 

In the first screening phase, the titles and abstracts of all of the articles identified in the search were screened, and studies that did not assess the efficacy or effectiveness of COVID-19 vaccines targeting emerging Omicron variants were excluded. In the second screening phase, full text articles of studies assessing the efficacy/effectiveness of the adapted COVID-19 vaccines were evaluated, and the following exclusion criteria were considered: (1) studies assessing the relative effectiveness of adapted vaccines; (2) insufficient explanation of the methodology (methods used, vaccines assessed, population included, study period, comparator definition); and (3) studies assessing the aVE in specific settings and population groups, such as individuals aged 65 years or more, children and groups of patients.

### 2.2. Assessment of the Ability of Adapted Vaccines to Establish Herd Immunity against Omicron Variants

Herd immunity can be established against an emerging SARS-CoV-2 variant when the prevalence of individuals protected against this variant (I) is higher than the critical prevalence, called “herd immunity threshold” (I > I_c_), thus blocking SARS-CoV-2 transmission in the population [5,9,32,33]. The herd immunity threshold in terms of the prevalence of protected individuals, I_c_, can be determined from the viral transmissibility or basic reproduction number, R_o_, defined as the average number of secondary cases generated by one primary case in a totally susceptible population:

I_c_ = 1 − (1/R_o_). (1)

The viral transmissibility is a primary driver of SARS-CoV-2 evolution because an emerging variant with greater transmissibility can spread more rapidly and substitute previously circulating variants [17,34]. Studies assessing the transmissibility of SARS-CoV-2 have found that the basic reproduction number, Ro, defined as the average number of secondary cases generated per infected case in a totally susceptible population during the infectious period, increased from 2.5 for the original wild-type variant to 6–12 for the Omicron variant [35,36,37,38,39,40]. Consequently, R_o_ values of 6 to 12 were assumed in this study for the Omicron BA.1, BA.2 and BA.4-5 variants. 

The ability of the available adapted vaccines to establish herd immunity against SARS-CoV-2 was assessed using two methods. The first method consisted in comparing the prevalence of vaccine-induced protected individuals generated by the adapted vaccines, determined from the vaccine effectiveness in preventing SARS-CoV-2 infection and the vaccination coverage (I_v_ = E × V), with the critical prevalence of protected individuals (I_c_). It was considered that the adapted vaccines could establish herd immunity against the Omicron BA.1, BA.2 and BA.4-5 variants if they were able to generate a prevalence of vaccine-induced protected individuals equal to or greater than the critical prevalence necessary to establish herd immunity against viruses with R_o_ values from 6 to 12 (I_v_ ≥ I_c_). 

The second assessment method consisted in determining the SARS-CoV-2 R_o_ values that the adapted vaccines could block, taking into account the following factors: (1) levels of vaccine effectiveness in preventing SARS-CoV-2 infection of 30%, 39% and 49%; (2) percentages of vaccination coverage from 60% to 100%; (3) use of NPIs to reduce viral transmissibility; and (4) prevalence of individuals already protected in the population of 0%, 10%, 20% and 30%. It was considered that the adapted vaccines could establish herd immunity against Omicron variants when they were able to block viruses with R_o_ values from 6 to 12.

In this study, the R_o_ values that the adapted vaccines could block were determined using the following formula:

R_o_ = 1/(1 − I_v_). (2)

When non-pharmaceutical interventions (NPIs) are used, SARS-CoV-2 transmissibility can be reduced and lower percentages of vaccination coverage are necessary to establish herd immunity in the population, depending on the NPIs’ effectiveness (ENPI) in reducing viral transmissibility. In this study, the R_o_ values that the adapted vaccines could block when NPIs are used were determined using the following formula:

R_o_ = (1/(1 − I_v_))/ENPI. (3)

The following values of effectiveness in reducing SARS-CoV-2 transmissibility were assumed in this study for NPIs: 15.1% for mandatory face masks; 38.6% for mandatory face masks and social distancing; 54.3% for mandatory face masks, social distancing and travel restrictions; and 62.8% for mandatory face masks, social distancing, travel restrictions and quarantine [28]. These values of effectiveness were obtained in a study that included 190 countries and was carried out during the COVID-19 pandemic [28]. 

When part of the population is already protected due to natural infection (I_n_), the vaccination coverage required to establish herd immunity is lower [5]. In this study, the R_o_ values that the adapted vaccines could block when NPIs are used and part of the population is already protected against an emerging SARS-CoV-2 variant were determined using the following formula:

R_o_ = [1/(1 − I_v_ − I_n_)]/ENPI. (4)

### 2.3. Challenges That Adapted Vaccines Should Overcome to Establish Herd Immunity against Emerging SARS-CoV-2 Variants

The challenges that vaccination programs using adapted vaccines should overcome in terms of vaccine effectiveness and vaccination coverage were assessed using two methods. The first method consisted in determining the critical vaccine effectiveness (E_c_) that adapted vaccines should achieve to establish herd immunity against emerging SARS-CoV-2 with R_o_ values from 6 to 12 for percentages of vaccination coverage (V) from 70% to 95%:

E_c_ = [1 − (1/R_o_)]/V. (5)

The vaccine effectiveness should be equal to or higher than the critical vaccine effectiveness (E ≥ E_c_) to establish herd immunity. This analysis was carried out in the following situations: (1) without using NPIs in the population; (2) using face masks; and (3) using face masks, social distancing and travel restrictions. 

The second method consisted in determining the critical vaccination coverage (V_c_) required to establish herd immunity against viruses with R_o_ values from 6 to 12 for levels of vaccine effectiveness (E) from 75% to 100%:

V_c_ = I_c_/E = [1 − (1/R_o_)]/E. (6)

The vaccination coverage should be equal to or higher than the critical coverage required to establish herd immunity against an emerging SARS-CoV-2 (V ≥ V_c_). This analysis was carried out in the following situations: (1) without using NPIs in the population; (2) using face masks; (3) using face masks, social distancing and travel restrictions; and (4) when a 10% prevalence of individuals are already protected due to natural infections in the population. 

### 2.4. Statistical Analysis

The Epidat program [41] was used to conduct a meta-analysis of the results obtained in the studies selected to obtain the overall OR for severe disease and for SARS-CoV-2 infection. Fixed-effects and random-effects meta-analyses were carried out. The overall aVE in preventing SARS-CoV-2 infection were estimated from: 1 − OR. The values of aVE that were considered outliers based on the IQR method were excluded. It was assumed that the aVE in preventing SARS-CoV-2 infection was 35% lower than the aVE in preventing severe disease, based on the results obtained by the Institute for Health Metrics and Evaluation (University of Washington) for COVID-19 vaccines [13]. The Chi-square test was used to assess whether the observed differences in the results included in the meta-analysis were compatible with chance alone (heterogeneity test), considering a *p* < 0.05 as statistically significant. The Begg test was used to test the publication bias, considering a *p* < 0.05 as statistically significant.

Microsoft Excel 2016 (v. 2203) was used to calculate the Ro values of SARS-CoV-2 that available adapted vaccines could block; the vaccine effectiveness that would be required to establish herd immunity against SARS-CoV-2 viruses with R_o_ values from 6 to 12; and the critical percentages of vaccination coverage that would be required to establish herd immunity for SARS-CoV-2 with Ro values of 6–12 and a vaccine effectiveness of 35–100%.

## 3. Results

### 3.1. Effectiveness of Adapted Vaccines in Preventing SARS-CoV-2 Infections

A PRISMA flow diagram was developed to present the selection process, as well as the number of articles identified, screened, excluded and selected (Supplementary Materials Figure S1). A total of 881 records were retrieved from the PubMed database. In the first screening phase, 866 articles that did not assess the efficacy/effectiveness of adapted vaccines targeting Omicron variants were excluded. In the second phase, 12 articles assessing the relative effectiveness of vaccines (rVE) targeting Omicron variants, one study assessing the aVE in preventing severe disease among individuals aged 65 years or more; two studies focusing on specific settings and population groups; and one study without a comparator definition were all excluded from the final selection. 

Three studies [22,23,24] had obtained eight aVE values ranging from 46% to 76% in preventing severe disease in the adult population (Table 1). No studies with aVE values in preventing SARS-CoV-2 infection were found in the systematic literature review. The overall risk of bias (ROBINS tool [31]) was considered low for the three studies selected.

The mean aVE was 59% and the median aVE was 60.5% (IQR: 49.3–66%). One of the aVE values (24%) was considered an outlier, based on the IQR method (lower than 1.5 IQR below Q_1_). The Chi-square test for heterogeneity was not significant. The Begg test assessing publication bias was not significant.

The overall OR comparing severe COVID-19 disease in individuals vaccinated with adapted vaccines and unvaccinated individuals was 0.44 in the fixed-effects meta-analysis and 0.40 in the random-effects meta-analysis (Table 1). The aVE in preventing severe disease was 56% in the random-effects meta-analysis and 60% in the random-effects meta-analysis (Table 1).

The values of aVE in preventing SARS-CoV-2 infection estimated from the results obtained in the selected studies ranged from 30% to 49% (Table 1). The aVE in preventing SARS-CoV-2 infection was 36% in the fixed-effects meta-analysis results and 39% in the random-effects meta-analysis results (Table 1). Consequently, the ability of the adapted vaccines in establishing herd immunity against SARS-CoV-2 was assessed for three values of aVE in preventing SARS-CoV-2 infection: 30%, 39% and 49%. 

### 3.2. Assessment of the Ability of Adapted Vaccines to Establish Herd Immunity against Omicron SARS-CoV-2 Variants

The ability of the adapted vaccines to establish herd immunity against the Omicron BA.1, BA.2 and BA.4-5 variants was very limited because they could generate a 36–39% prevalence of vaccine-induced protected individuals, assuming 100% vaccination coverage, which was lower than the 83–92% prevalence of protected individuals necessary to establish herd immunity against SARS-CoV-2 with R_o_ values from 6 to 12. 

Table 2 presents the herd immunity assessment for the adapted vaccines, based on the SARS-CoV-2 R_o_ values they were able to block in different situations.

Table 2 shows that the ability of the adapted vaccines to establish herd immunity against the Omicron BA.1, BA.2 and BA.4-5 variants was very limited due to their 30–49% effectiveness in preventing SARS-CoV-2 infection. Nevertheless, the adapted vaccines were able to establish herd immunity against viruses with R_o_ values from 6 to 12 when the most effective combination of NPIs (face masks, social distancing, travel restrictions and quarantine) was used in a population with a 10–30% prevalence of individuals already protected due to natural infection. For example, the adapted vaccines were able to establish herd immunity against viruses with R_o_ values of 6–9 by combining 39–49% vaccine effectiveness, 95–100% vaccination coverage and ≥10% prevalence of protected individuals; and against viruses with R_o_ values of 10–12 by combining 49% vaccine effectiveness, 90–100% vaccination coverage and ≥30% prevalence of protected individuals (Table 2).

### 3.3. Challenges That Adapted Vaccines Should Overcome to Establish Herd Immunity against Emerging SARS-CoV-2 Variants

Table 3 presents the levels of vaccine effectiveness in preventing SARS-CoV-2 infection that adapted vaccines should achieve to establish herd immunity against SARS-CoV-2 with R_o_ values from 6 to 12, with percentages of vaccination coverage from 70% to 95%, without using NPIs and with using NPIs in the population. Without using NPIs in the population, vaccine effectiveness should be 88–100% and vaccination coverage should be 90–95% (Table 3). Vaccination coverage objectives of 90% and 95% would be sufficient to establish herd immunity against viruses with R_o_ values of 6–10 and 6–12, although the vaccine effectiveness should be greater than 88% and 93%, respectively (Table 3). 

The levels of vaccine effectiveness that adapted vaccines should achieve to establish herd immunity were lower when NPIs were used in the population. Using face masks in the population, vaccine effectiveness should be 85–97% to establish herd immunity against viruses with R_o_ values of 6–9 and 92–100% against viruses with R_o_ values of 9.5–12, and the vaccination coverage should be 90–95% (Table 3). 

Using face masks and social distancing in the population, the vaccine effectiveness should be 77–99.6% to establish herd immunity against viruses with R_o_ values of 6–9, and 87–96% against viruses with R_o_ values of 9.5–12, and the vaccination coverage should be 80–95% (Table 3). 

Using face masks, social distancing and travel restrictions in the population, the vaccine effectiveness should be 67–98% to establish herd immunity against viruses with R_o_ values of 6–9, and 81–99% against viruses with an R_o_ of 9.5–12 (Table 3). 

Using face masks, social distancing, travel restrictions and quarantine in the population, the vaccine effectiveness should be 58–98% to establish herd immunity against viruses with R_o_ values of 6–9, and 75–97% against viruses with R_o_ values of 9.5–12 (Table 3).

**Figure 1:**
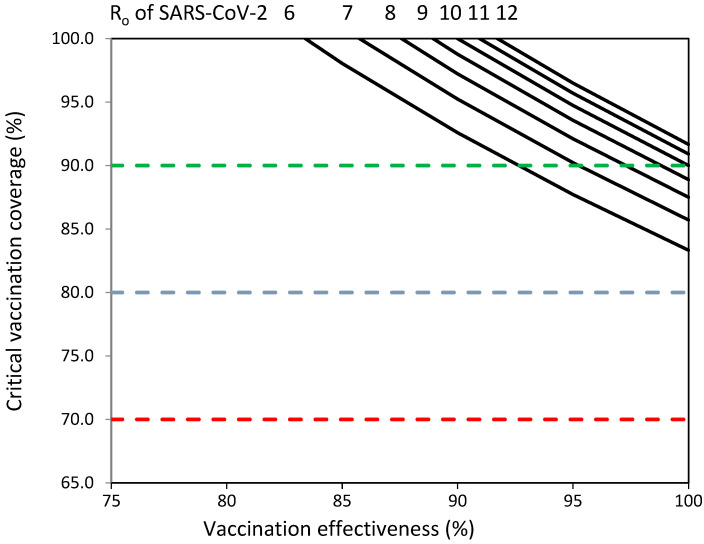
Vaccination coverage (%) required to establish herd immunity against SARS-CoV-2 with reproductive numbers (R_o_) from 6 to 12 by effectiveness (%) of adapted SARS-CoV-2 vaccines. Objectives of vaccination coverage of 70%, 80% and 90% indicated with dashed red, blue and green lines, respectively.

Figure 1 shows that adapted vaccines should overcome important challenges in terms of vaccine effectiveness and vaccination coverage to establish herd immunity against emerging SARS-CoV-2 variants with R_o_ values from 6 to 12. COVID-19 vaccination programs should achieve percentages of vaccination coverage greater than 80% against emerging SARS-CoV-2 variants with R_o_ values from 6 to 12 (Figure 1). Vaccination coverage objectives of 70% and 80% could not be sufficient to establish herd immunity against emerging variants with R_o_ values from 6 to 12 (Figure 1). Adapted vaccines should achieve >85% vaccination coverage and >80% vaccine effectiveness to establish herd immunity against viruses with R_o_ values of 6–7; >85% vaccination coverage and 87% vaccine effectiveness to establish herd immunity against viruses with R_o_ values of 8–9; and >90% vaccination coverage and >90% vaccine effectiveness to establish herd immunity against viruses with R_o_ values of 10–12 (Figure 1). 

The challenges in terms of vaccine effectiveness and vaccination coverage that adapted vaccines should overcome to establish herd immunity against emerging SARS-CoV-2 variants with R_o_ values from 6 to 12 were less difficult when NPIs were used in the population (Supplementary Materials Figures S2 and S3) and when NPIs were used in a population with 10% of the population already protected against an emerging variant due to natural infection (Supplementary Materials Figures S4 and S5). 

## 4. Discussion

This study found that the adapted vaccines were associated with aVE values of 56–60% in preventing severe disease, aVE values of 36–39% in preventing SARS-CoV-2 infection and a limited ability to establish herd immunity against the Omicron BA.1 and BA.4-5 variants. When COVID-19 vaccines were available in 2021, it was expected that achieving a 70% vaccination coverage could be sufficient to establish anti-SARS-CoV-2 herd immunity in the population [9,10]. Nevertheless, this study found that the estimated effectiveness in preventing SARS-CoV-2 infection of the adapted vaccines was not sufficient to generate the prevalence of vaccine-induced protected individuals required to block the transmission of viruses with R_o_ values from 6 to 12. With a 36–39% effectiveness in preventing infection, the adapted vaccines could not generate the 83–92% prevalence of protected individuals necessary to establish herd immunity against SARS-CoV-2.

The values of aVE in preventing SARS-CoV-2 infection achieved with the adapted vaccines found in this study were lower than those achieved with prior mRNA vaccines against infections caused by the wild-type virus (86–92%) and the Delta variant (84–91%) [13]. Lower levels of VE in preventing symptomatic infection caused by the Omicron BA.1 and Omicron BA.2 variants before August 2022 (49–53%) were found in studies assessing the VE of booster vaccinations using vaccines targeting the wild-type virus [15]. 

The lower effectiveness in preventing Omicron infection of the available adapted vaccines can be explained by the following factors: (1) higher viral transmissibility due to higher binding affinity between the SARS-CoV-2 receptor binding domain (RBD) and the human angiotensin-converting enzyme isoenzyme 2 (ACE-2) receptor due to mutations located at the RBD [42,43,44,45]; (2) a greater ability to infect nasopharyngeal and bronchial cells and lesser ability to infect bronchial and lung cells (cellular tropism changes) than the wild-type SARS-CoV-2 [39,42]; (3) a shorter incubation period between the infection and the moment an infected individual become infectious to other individuals [17]; and (4) a higher immune escape from vaccine-induced and natural infection immunity [14,42,43,44,45]. 

Immune escape is defined as the failure of humoral or cellular immunity to recognize and neutralize a virus derived from its acquired mutations. Immune escape is a primary driver of SARS-CoV-2 evolution because emerging viruses resistant to vaccine-induced and natural infection immunity responses will be able to substitute less resistant viruses [17,42]. There is a direct relation between the immune escape and higher reinfection rates observed during the Omicron wave among individuals protected against prior variants [46,47]. The consequence is a lower level of effectiveness in preventing infection for the adapted vaccines than for prior vaccines targeting the wild-type virus.

Neutralizing antibodies bind to the pathogenic surface proteins that mediate adhesion to the host cell and intracellular entry [42]. Vaccine-induced anti-RBD antibodies generated by the adapted vaccines can be less effective against the BA.1 and BA.4-5 Omicron variants than those generated by prior vaccines against prior variants, because the Omicron variants have acquired mutations in the RBD associated with reduced antibody neutralization activity [48]. Nevertheless, bivalent BA.1 and BA.4/BA.5 booster vaccines are able to generate higher Omicron-neutralization titers than monovalent booster vaccines targeting the wild-type virus [49]. 

An insufficient mucosal immunity protection against Omicron variants can be associated with lower levels of effectiveness at preventing infection because mucosal immunity has a critical role in protecting mucosal surfaces against respiratory viruses [50]. A study that assessed the humoral immune response in patients with severe COVID-19 disease found that IgA-mediated mucosal immunity may be a critical defense mechanism against SARS-CoV-2 at the individual level that may reduce the infectivity of human secretions and viral transmission [51].

The higher transmissibility of the Omicron variants compared with prior variants can be explained by several factors: (1) higher binding affinity between the SARS-CoV-2 RBD and the ACE-2 receptor; (2) greater ability to infect nasopharyngeal and bronchial cells than the wild-type variant; (3) viral immune escape; and (4) shorter latent or incubation period between infection and the moment an infected individual becomes infectious. A higher RBD-ACE-2 binding affinity is associated with a higher virus transmissibility because a more efficient viral entry into the human respiratory cells results in a faster infective cycle and increased viral production from infected individuals, as well as an increased ability to infect susceptible individuals [17,52]. The viral production from infected individuals is higher when the RBD-ACE2 binding affinity increases, because the number of infected cells is higher and the viral production cycle is faster [52]. The ability to infect susceptible individuals is higher when the RBD-ACE2 binding affinity increases because a higher viral-ACE2 affinity is associated with a higher viral load in mucosal secretions of infected individuals [17,52].

Could the adapted vaccines establish herd immunity using NPIs in the population? The study found that the improvement in the ability of the adapted vaccines to establish herd immunity using NPIs was very limited. NPIs reduced viral transmissibility by 15% to 63%, depending on the combination of measures used in the population, but the adapted vaccines were still not able to generate sufficient herd immunity against SARS-CoV-2 variants with greater transmissibility. With a 0% prevalence of individuals already protected in the population, the adapted vaccines could establish herd immunity against SARS-CoV-2 with Ro values of 4–5, but not against viruses with R_o_ values from 6 to 12. The adapted vaccines could establish herd immunity against viruses with R_o_ values from 6 to 12 when only using highly effective NPIs in a population with a 20–30% prevalence of individuals already protected. 

A study carried out in Japan found that the population immunity levels required for the transient suppression of symptomatic infections in the fifth, sixth and seventh COVID-19 waves in three Japanese prefectures (Tokyo, Osaka, Aichi) ranged from 35% to 45% for the entire population and from 20% to 40% for the population aged 10–64 years [53]. These thresholds were lower than those obtained in this study for preventing SARS-CoV-2 transmission, based on the herd immunity model. Nevertheless, it is difficult to compare the results obtained in this study with those obtained in the Japanese study due to the considered outcomes and methodological differences. The study carried out in Japan was focused on determining the population immunity levels that reduced COVID-19 daily symptomatic infections for different waves, using data reported for daily vaccinations and daily symptomatic infections, and taking into account assumptions for the rate of asymptomatic infections and vaccine effectiveness against different SARS-CoV-2 variants. The population immunity levels associated with the transient suppression of symptomatic infection found in the Japanese study cannot be compared with the percentages of vaccinated and protected individuals blocking SARS-CoV-2 transmission based on the herd immunity theory due to (1) differences between herd immunity against SARS-CoV-2 transmission and immunity level for transient suppression of symptomatic infection; (2) the greater effectiveness of COVID-19 vaccines in preventing symptomatic infections than in preventing SARS-CoV-2 transmission and; (3) population immunity levels were determined in the Japanese study using reported data on daily confirmed cases and daily vaccinations. 

The ability of COVID-19 vaccines to establish herd immunity against SARS-CoV-2 had been questioned because the phenotypic stability of SARS-CoV-2 is lower than that of measles and polio viruses, and the duration of vaccine-induced and natural immunity against SARS-CoV-2 is lower than for measles and polio viruses [11]. The development of adapted vaccines targeting new emerging SARS-CoV-2 variants and booster vaccinations every 6–12 months could solve these limitations. Nevertheless, this study found that COVID-19 vaccination programs must overcome several challenges to create herd immunity against emerging SARS-CoV-2 variants. Firstly, the effectiveness of adapted vaccines in preventing infection must be >80%. Secondly, vaccine access must be increased to achieve percentages of vaccination coverage higher than 70%. 

In the new socio-economic normality, the COVID-19 vaccination strategy implemented in each country depends on several factors, including the public health impact of emergent variants, the efficacy and effectiveness of vaccines in preventing severe disease, vaccination coverage objectives, waning immunity and the ability of vaccines to establish herd immunity and block SARS-CoV-2 transmission. The COVID-19 vaccination coverage objective could be decided based on the critical vaccination coverage required to block SARS-CoV-2 transmission in the population [5], but the ability of adapted vaccines to establish herd immunity against new emerging variants depends on the development of adapted vaccines with sufficient effectiveness in preventing SARS-CoV-2 infection. 

The European Medicines Agency (EMA) and other agencies approved the marketing authorization of COVID-19 vaccines based on their safety and efficacy results obtained in phase 3 clinical trials involving adults, high-risk individuals and individuals aged 65 years or more [54]. The primary endpoint in pivotal vaccine efficacy trials should be laboratory-confirmed COVID-19 disease of any severity, and secondary endpoints should include estimates of the protection against symptomatic and severe disease [54]. The EMA considered that values of vaccine efficacy of at least 50% and a lower bound of the 95% confidence interval around the point estimate above 20–30% would be sufficient to provide a convincing demonstration of vaccine efficacy [54]. Nevertheless, this study found that these values of VE are not sufficient to prevent SARS-CoV-2 transmission in the population. Adapted vaccines targeting the BA.1 and BA.4-5 variants were approved based on their higher immunity activity against the Omicron variants than vaccines targeting the wild-type virus [55,56]. The EU’s strategy is to have a broad range of adapted vaccines that target different SARS-CoV-2 variants, so that different countries can have a plurality of options with which to design their vaccination strategies [55]. New vaccines should be developed to achieve higher levels of vaccine effectiveness at preventing severe diseases and SARS-CoV-2 infection.

After declaring on 5 May 2023 that COVID-19 is no longer a global health emergency, the WHO Europe proposed the development of a Transition Plan for COVID-19 with the objective to strengthen health emergency preparedness, response and resilience by means of implementing five core strategies [7]: (1) collaborative surveillance; (2) reviewing and updating community protection measures; (3) investing in health services and emergency care systems; (4) considering COVID-19 vaccination as part of wider immunization activities; and (5) coordination between COVID-19 and influenza surveillance and control activities and those against other respiratory viruses. 

Most countries are moving towards COVID-19 vaccination strategies consisting of regular booster vaccinations once per year to target population groups using updated adapted vaccines against currently circulating viruses. In this strategy, COVID-19 vaccinations can be administered annually before the SARS-CoV-2 season, like the influenza virus vaccination campaigns. The target population groups include vulnerable individuals, adults and children, and vaccination decisions should be revised by national and international committees. Nevertheless, the seasonal COVID-19 vaccination strategy including only vulnerable population groups cannot establish herd immunity in the population [5], and this vaccination strategy is adequate only if SARS-CoV-2 infections follow a seasonal pattern.

If SARS-CoV-2 infections occur during the year with small or big waves of infection due to new emergent variants, the prevention and control strategy should include the following activities:

(1) Detect the emergent variants.
(2) Assess the health impact of the emergent variants.
(3) Monitor and characterize the transmissibility and immune escape of the emergent variants.
(4) Assess the anti-SARS-CoV-2 immunity levels in the population.
(5) Assess the percentages of COVID-19 vaccination coverage required to establish herd immunity.
(6) Decide the vaccination strategy to protect vulnerable population groups and to prevent SARS-CoV-2 transmission in the population.
(7) Develop COVID-19 booster vaccinations to overcome waning vaccine-induced immunity.
(8) Develop updated and new vaccines against emergent variants of concern to increase COVID-19 vaccination effectiveness in preventing severe disease and SARS-CoV-2 infection.
(9) Use non-pharmacological measures if they are necessary to reduce viral transmissibility among vulnerable population groups and to reduce SARS-CoV-2 transmission in the population.

Monitoring the transmissibility and immune escape of emerging variants is necessary to assess the public health threat caused by their genomic diversity, and to assess potential vaccine effectiveness reductions [48,57,58,59,60]. Monitoring anti-SARS-CoV-2 immunity levels in the population and vulnerable population groups is necessary to assess the risk of SARS-CoV-2 transmission and severe COVID-19 and to estimate the percentages of COVID-19 vaccination coverage required to prevent SARS-CoV-2 transmission [5]. 

COVID-19 vaccination programs using adapted vaccines must include the whole population (universal vaccination) and develop intensive vaccination activities targeting specific population groups with lower levels of population immunity to achieve percentages of vaccination coverage higher than 80–85%. Vaccination programs targeting only the elderly and vulnerable individuals cannot establish herd immunity in the whole population [5]. In a stratified universal vaccination strategy, different age groups can be immunized at different phases, including vulnerable population groups in the first phase and other adults, children and adolescents in subsequent phases [5]. COVID-19 vaccination programs must include children and adolescents because SARS-CoV-2 can be transmitted in populations with low levels of anti-SARS-CoV-2 immunity among children and adolescents [5,19,61]. The overall values of vaccine effectiveness were used in this study to estimate the percentages of vaccination coverage necessary to establish herd immunity in the population, although greater percentages should be achieved among population groups with lower levels of vaccine effectiveness. 

This study has several limitations. Firstly, the herd immunity assessment for the adapted vaccines used a herd immunity model where the establishment of herd immunity depends on SARS-CoV-2 transmissibility and vaccine effectiveness. This method is based on the following assumptions: (1) a homogeneous mixing of individuals within the population and (2) a homogeneous distribution of protected individuals within the population [9]. Nevertheless, it is possible to assume a homogeneous mixing of persons and a homogeneous distribution of protected individuals within vaccinated population groups [5]. Secondly, the herd immunity analysis was carried out assuming R_o_ values for SARS-CoV-2 from 6 to 12. Values of R_o_ higher than 12 would make it more difficult to establish herd immunity. Thirdly, the effectiveness in preventing SARS-CoV-2 infection of 36–39% estimated in this study for the adapted vaccines was determined from the absolute effectiveness at preventing severe disease obtained in three studies selected from a systematic literature review. The effectiveness may be higher or lower than that assumed in this study when more studies are developed. Nevertheless, this study assessed the ability of the adapted vaccines to establish herd immunity for a range of vaccine effectiveness levels to overcome this limitation.

## 5. Conclusions

This study found that the adapted vaccines targeting BA.1 and BA.4-5 have a limited ability to establish herd immunity against the BA.1 and BA.4-5 Omicron variants due to their 36–39% effectiveness in preventing SARS-CoV-2 infection. The adapted vaccines could not establish herd immunity against the Omicron BA.1 and BA.4-5 variants without the use of NPIs. The adapted vaccines could establish herd immunity only by achieving >80% vaccination coverage, using NPIs with greater effectiveness and with a 20–30% prevalence of protected individuals in the population. New, adapted COVID-19 vaccines with greater effectiveness in preventing SARS-CoV-2 infection should be developed to establish herd immunity against emerging SARS-CoV-2 variants

## Figures and Tables

**Table 1:** Absolute effectiveness (aVE) in preventing SARS-CoV-2 infection of the adapted vaccines targeting Omicron BA1 and BA.4-5 variants estimated from the meta-analysis of results obtained in studies selected.

Vaccine	Study Population, Country	Outcome	No. Positive/No. Negative	aVE % (95% CI)
Link-Gelles et al. [22]				
Bivalent vaccine	Adults USA	Hospitalization 7–59 days after vaccination	unV: 1791/13723 V: 327/4530	62 (57–67)
		Hospitalization 60–115 days after vaccination	unV: 1791/13723 V:486/4705	47 (41–53)
		Critical care 7–59 days after vaccination	unV: 367/13723 V: 49/4530	69 (57–77)
		Critical care 60–115 days after vaccination	unV: 367/13723 V: 85/4705	46 (30–58)
		Critical care 120–179 days after vaccination	unV: 367/13723 V: 33/2995	50 (26–66)
Tendorde et al. [23]				
Bivalent vaccine	Adults USA	Hospitalization ≥7 days after vaccination	unV: 434/3658V: 56/828	59 (44–70)
Arashiro et al. [24]				
BA.1 vaccine	Adults Japan	Hospitalization ≥14 days after vaccination	unV: 442/226 V: 95/76	65 (47–77)
BA.4-5 vaccine			unV: 442/226 V: 112/116	76 (65–83)
Overall aVE in preventing severe disease obtained via meta-analysis				
Range of values from selected studies: 46–76%				
Fixed-effects meta-analysis: 56% (95% CI: 53–59%) Random-effects meta-analysis: 60% (95% CI: 51–66%)				
aVE in preventing SARS-CoV-2 infection (35% lower than that for severe disease [13])				
Range of values from selected studies: 30–49%				
Estimated from the fixed-effects meta-analysis results: 36% (95% CI: 34–38%) Estimated from the random-effects meta-analysis results: 39% (95% CI: 33–43%)				

V: Vaccinated individuals. uV: unvaccinated individuals.

**Table 2:** R_o_ values of SARS-CoV-2 that adapted vaccines targeting Omicron BA.1 and BA.4-5 variants could block for levels of vaccine effectiveness of 30%, 39% and 49% and percentages of vaccination coverage from 60% to 100% in the following situations: (1) without using NPIs and with 0% prevalence of individuals already protected against SARS-CoV-2; (2) using NPIs in the population; (3) using the most effective combination of NPIs (MDTQ) in a population with a prevalence of protected individuals of 10%, 20% and 30%.

Effectiveness of Adapted Vaccines (%)	R_o_ Values of SARS-CoV-2 that Adapted Vaccines Could Block					
	Vaccination Coverage					
	60%	70%	80%	90%	95%	100%
(1) Without using NPIs and 0% prevalence of protected individuals in the population (0P)						
30	1.22	1.27	1.32	1.37	1.40	1.43
39	1.31	1.38	1.45	1.54	1.59	1.64
z	1.42	1.52	1.64	1.79	1.87	1.96
(2) Using face masks and 0P						
30	1.44	1.49	1.55	1.61	1.65	1.68
39	1.54	1.62	1.71	1.81	1.87	1.93
49	1.67	1.79	1.94	2.11	2.20	2.31
Using face masks and social distancing and 0P						
30	1.99	2.06	2.14	2.23	2.28	2.33
39	2.13	2.24	2.37	2.51	2.59	2.67
49	2.31	2.48	2.68	2.91	3.05	3.19
Using face masks, social distancing, travel restrictions and 0P						
30	2.67	2.77	2.88	3.00	3.06	3.13
39	2.86	3.01	3.18	3.37	3.48	3.59
49	3.10	3.33	3.60	3.91	4.09	4.29
Using face masks, social distancing, travel restrictions, quarantine (MDTQ) and 0P						
30	3.28	3.40	3.54	3.68	3.76	3.84
39	3.51	3.70	3.91	4.14	4.27	4.41
49	3.81	4.09	4.42	4.81	5.03	5.27
(3) Using NPIs (MDTQ) and 10% prevalence of protected individuals in the population						
30	3.73	3.90	4.07	4.27	4.37	4.48
39	4.04	4.29	4.57	4.90	5.08	5.27
49	4.44	4.83	5.29	5.86	6.19	6.56
Using MDTQ and 20% prevalence of protected individuals in the population						
30	4.34	4.56	4.80	5.07	5.22	5.38
39	4.75	5.10	5.51	5.99	6.26	6.56
49	5.31	5.88	6.59	7.49	8.04	8.67
Using MDTQ and 30% prevalence of protected individuals in the population						
30	5.17	5.49	5.84	6.25	6.48	6.72
39	5.77	6.30	6.93	7.70	8.16	8.67
49	6.62	7.53	8.73	10.38	11.46	12.80

NPI: non-pharmacological interventions.

**Table 3:** Effectiveness in preventing SARS-CoV-2 infection for adapted vaccines required to establish herd immunity against emerging SARS-CoV-2 variants with Ro values from 6 to 12, percentages of vaccination coverage from 60% to 95%, and using non-pharmacological interventions in the population.

Ro Value for SARS-CoV-2	Vaccine Effectiveness (%) ^a^ in Preventing SARS-CoV-2 Infection Required to Establish Herd Immunity Against Emerging SARS-CoV-2 Variants with R_o_ from 6 to 12 with Percentages of Vaccination Coverage from 70% to 95%															
	Without Using NPIs ^b^		Using Face Masks ^b^		Using Face Masks and Social Distancing ^b^				Using Face Masks, Social Distancing and Travel Restrictions				Using Face Masks, Social Distancing, Travel Restrictions and Quarantine			
	90%	95%	90%	95%	70%	80%	90%	95%	70%	80%	90%	95%	70%	80%	90%	95%
6	92.6	87.7	89.3	84.6	–	91.1	81.0	76.7	90.8	79.4	70.6	66.9	78.9	69.0	61.3	58.1
6.5	94.0	89.1	91.0	86.2	–	93.7	83.3	78.9	94.8	82.9	73.7	69.8	83.8	73.3	65.2	61.7
7	95.2	90.2	92.4	87.6	–	95.9	85.3	80.8	98.2	85.9	76.4	72.4	88.0	77.0	68.4	64.8
7.5	96.3	91.2	93.7	88.7	–	97.9	87.0	82.4	–	88.5	78.7	74.6	91.7	80.2	71.3	67.5
8	97.2	92.1	94.8	89.8	–	99.6	88.5	83.8	–	90.8	80.7	76.5	94.9	83.0	73.8	69.9
8.5	98.0	92.9	95.7	90.7	–	–	89.8	85.1	–	92.8	82.5	78.2	97.7	85.5	76.0	72.0
9	98.8	93.6	96.6	91.5	–	–	91.0	86.2	–	94.6	84.1	79.7		87.7	77.9	73.8
9.5	99.4	94.2	97.3	92.2	–	–	92.1	87.2	–	96.2	85.5	81.0	–	89.6	79.7	75.5
10	100.0	94.7	98.0	92.9	–	–	93.0	88.1	–	97.6	86.8	82.2	–	91.4	81.2	77.0
10.5	–	95.2	98.6	93.5	–	–	93.9	88.9	–	99.0	88.0	83.3	–	93.0	82.7	78.3
11	–	95.7	99.2	94.0	–	–	94.7	89.7	–	–	89.0	84.3	–	94.5	84.0	79.5
11.5	–	96.1	99.7	94.5	–	–	95.4	90.4	–	–	90.0	85.2	–	95.8	85.1	80.7
12	–	96.5	–	94.9	–	–	96.0	91.0	–	–	90.9	86.1	–	97.0	86.2	81.7

^a^: Vaccine effectiveness = (1 − (1/Ro))/vaccination coverage. The “–“ term indicates that adapted vaccines could not establish herd immunity with 100% vaccination coverage. ^b^: The adapted vaccines could not establish herd immunity with percentages of vaccination coverage of 70% and 80% without using NPIs and using face masks in the population, and with 70% vaccination coverage using face masks and social distancing in the population.

## Data Availability

Data are not publicly available. Data sharing is possible, subject to its request and approval.

## Supplementary Materials

The following are available online at https://www.mdpi.com/article/10.3390/vaccines11121836/s1: Figure S1: PRISMA flow diagram. Figure S2: Vaccination coverage (%) required to establish herd immunity against SARS-CoV-2 with reproductive numbers (R_o_) from 6 to 12 by effectiveness (%) for adapted vaccines using face masks to reduce viral transmissibility by 15.1%. Objectives of vaccination coverage of 70%, 80% and 90% indicated by dashed red, blue and green lines, respectively. Figure S3: Vaccination coverage (%) required to establish herd immunity against SARS-CoV-2 with reproductive numbers (R_o_) from 6 to 12 by effectiveness (%) for adapted vaccines using face masks, social distancing and travel restrictions to reduce viral transmissibility by 54.3%%. Objectives of vaccination coverage of 70%, 80% and 90% indicated by dashed red, blue and green lines, respectively. Figure S4: Vaccination coverage (%) required to establish herd immunity against SARS-CoV-2 with reproductive numbers (R_o_) from 6 to 12 by effectiveness (%) for adapted vaccines using face masks to reduce viral transmissibility by 15.1%. With 10% prevalence of protected individuals in the population. Objectives of vaccination coverage of 70%, 80% and 90% indicated by dashed red, blue and green lines, respectively. Figure S5: Vaccination coverage (%) required to establish herd immunity against SARS-CoV-2 with reproductive numbers (R_o_) from 6 to 12 by effectiveness (%) for adapted vaccines using face masks, social distancing and travel restrictions to reduce viral transmissibility by 54.3%%. With 10% prevalence of protected individuals in the population. Objectives of vaccination coverage of 70%, 80% and 90% indicated by dashed red, blue and green lines, respectively.
