# Supplementary material for: Effectiveness of Adapted COVID-19 Vaccines and Ability to Establish Herd Immunity against Omicron BA.1 and BA4-5 Variants of SARS-CoV-2
Source: Vaccines (Basel). 2023 Dec 10;11(12):1836. doi: 10.3390/vaccines11121836 (PMC10747774; doi:10.3390/vaccines11121836)

Supplementary material

Supplementary Figure S1. Prisma flow diagram

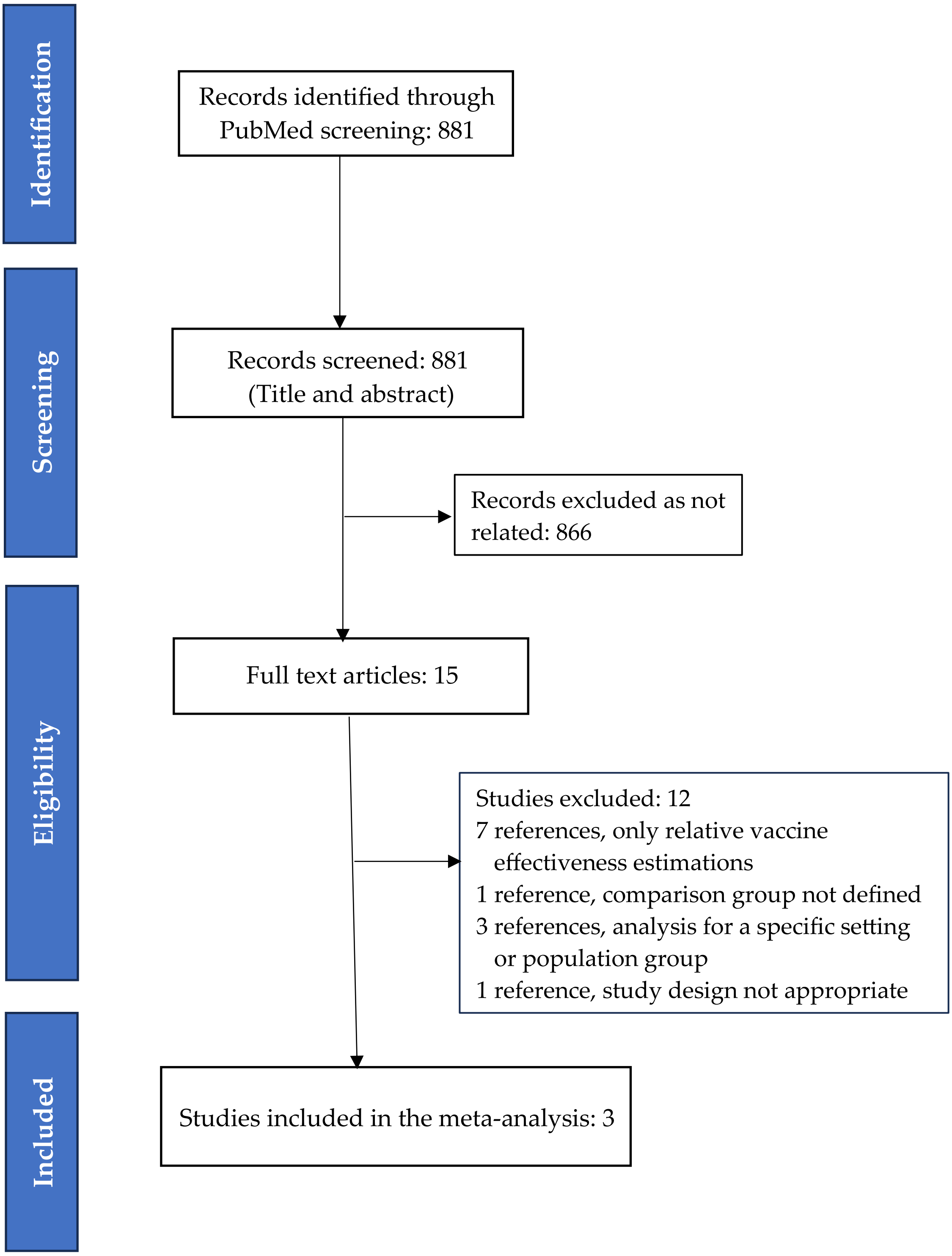

Supplement Figure S2. Vaccination coverage (%) required to establish herd immunity against SARS-CoV-2 with reproductive numbers (R_o) from 6 to 12 by effectiveness (%) for adapted vaccines using face masks to reduce viral transmissibility by 15.1%. Objectives of vaccination coverage of 70%, 80% and 90% indicated by dashed red, blue and green lines, respectively

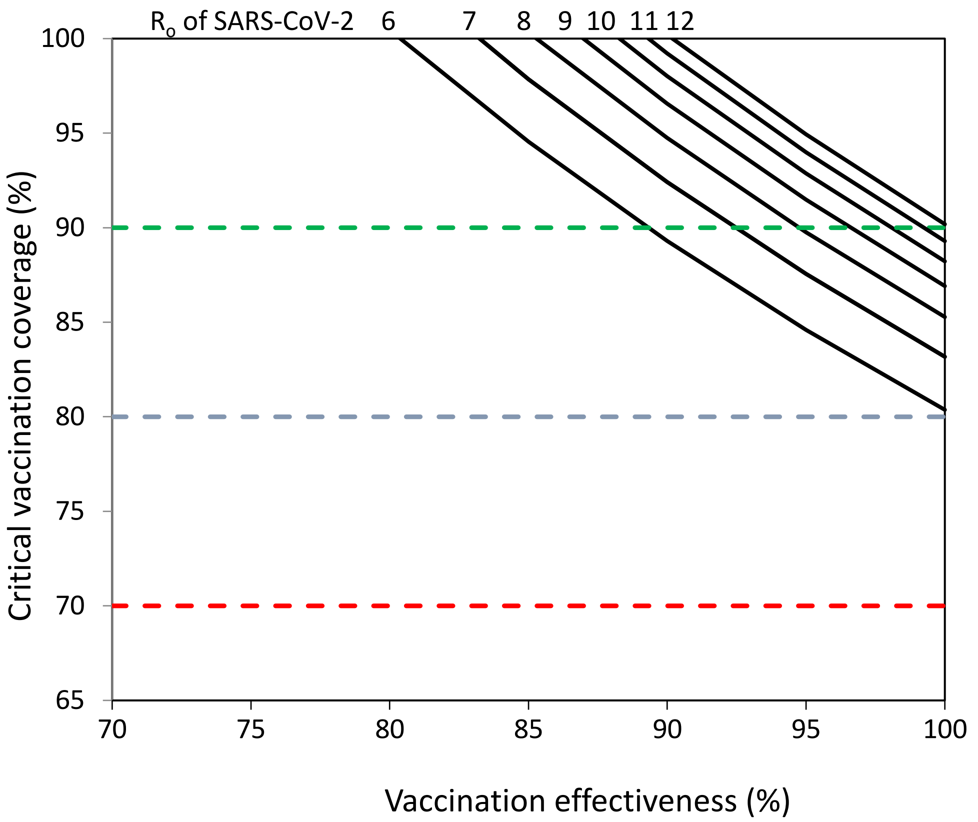

Supplement Figure S3. Vaccination coverage (%) required to establish herd immunity against SARS-CoV-2 with reproductive numbers (R_o) from 6 to 12 by effectiveness (%) for adapted vaccines using face masks, social distancing and travel restrictions to reduce viral transmissibility by 54.3%%. Objectives of vaccination coverage of 70%, 80% and 90% indicated by dashed red, blue and green lines, respectively

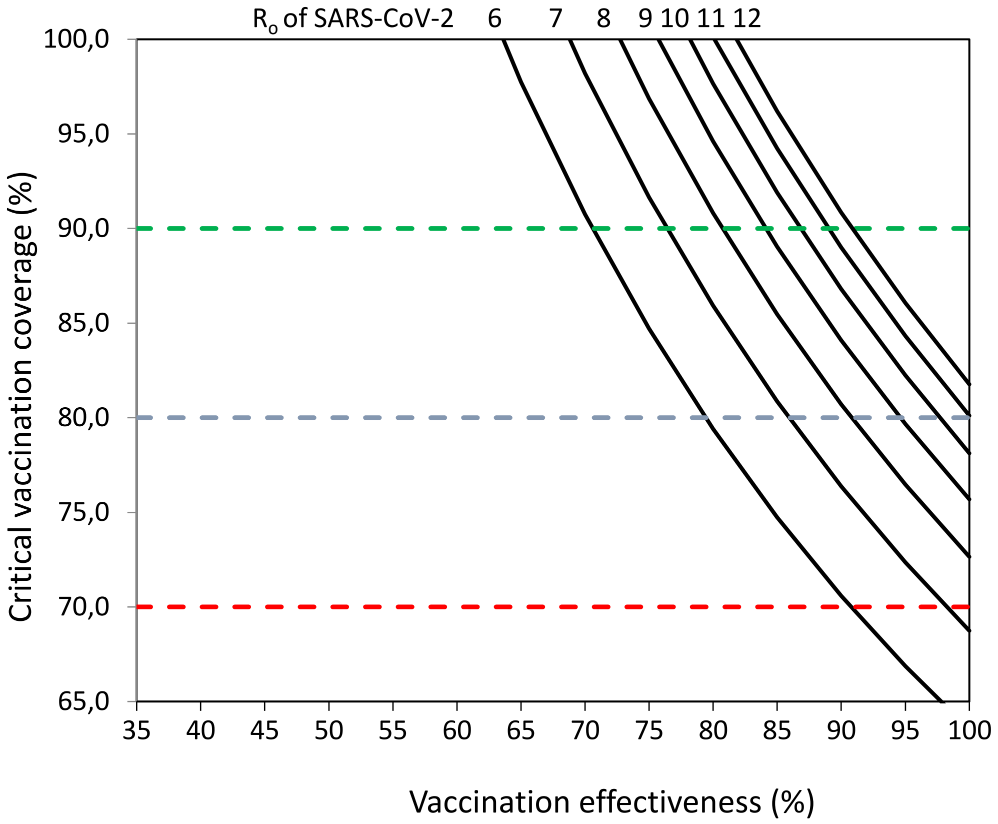

Supplement Figure S4. Vaccination coverage (%) required to establish herd immunity against SARS-CoV-2 with reproductive numbers (R_o) from 6 to 12 by effectiveness (%) for adapted vaccines using face masks to reduce viral transmissibility by 15.1%. With 10% prevalence of protected individuals in the population. Objectives of vaccination coverage of 70%, 80% and 90% indicated by dashed red, blue and green lines, respectively

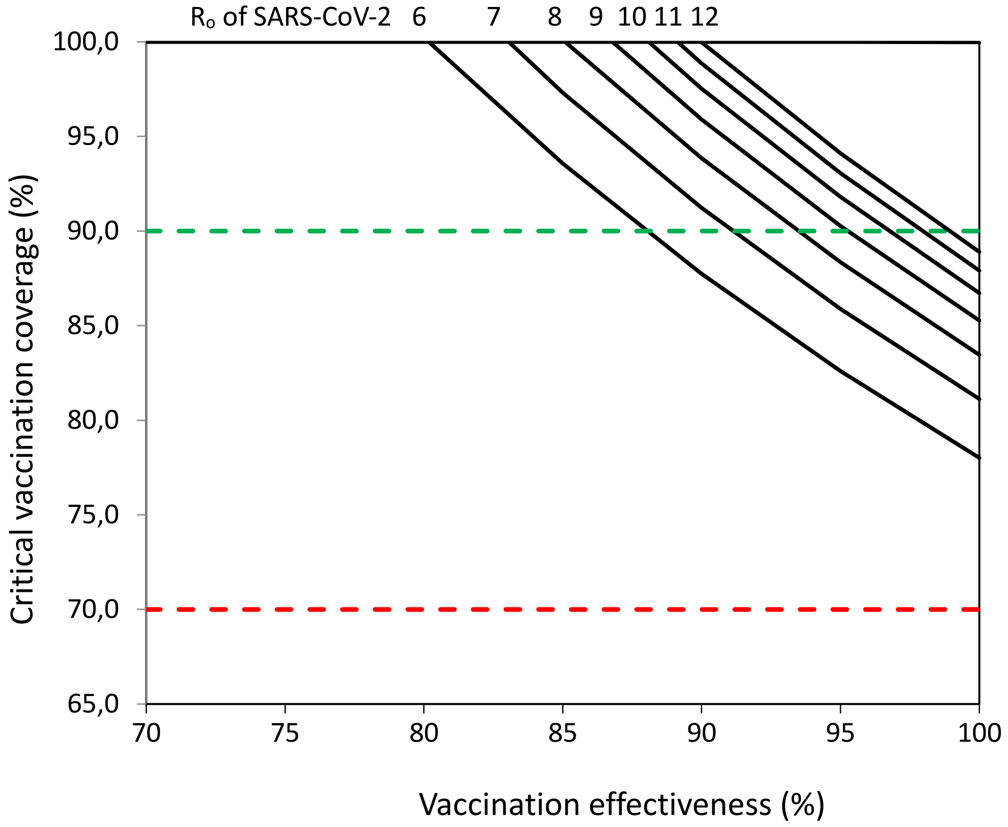

Supplement Figure S5. Vaccination coverage (%) required to establish herd immunity against SARS-CoV-2 with reproductive numbers (Rₒ) from 6 to 12 by effectiveness (%) for adapted vaccines using face masks, social distancing and travel restrictions to reduce viral transmissibility by 54.3%%. With 10% prevalence of protected individuals in the population. Objectives of vaccination coverage of 70%, 80% and 90% indicated by dashed red, blue and green lines, respectively

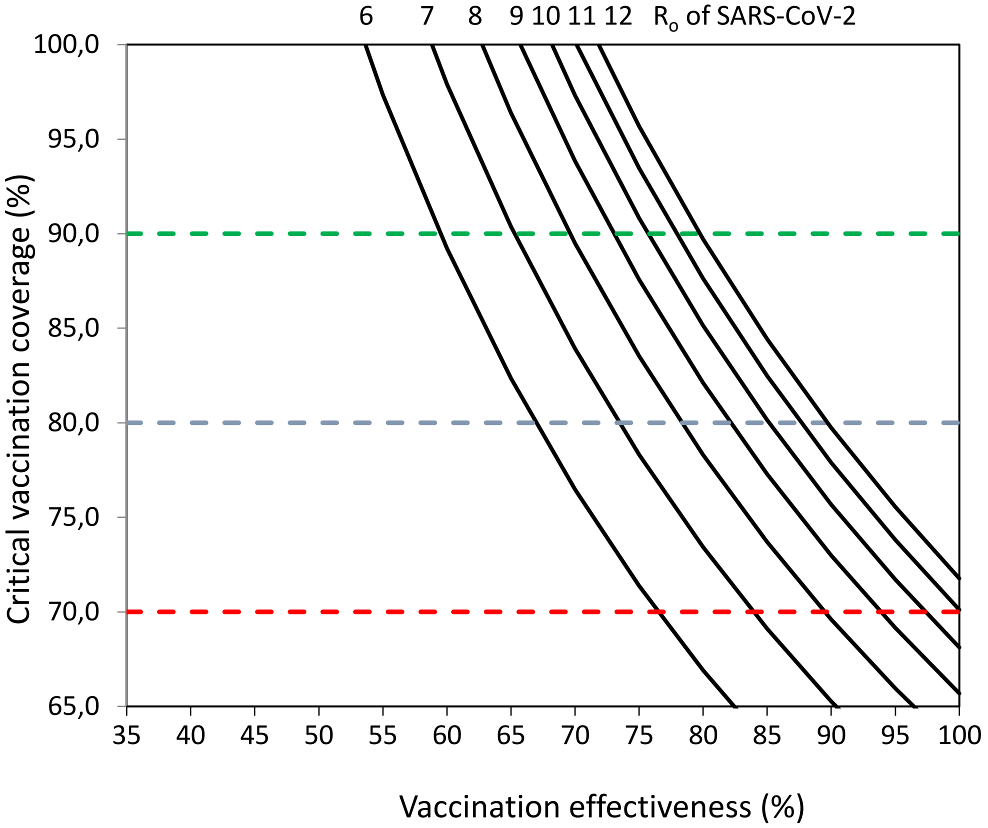

Supplement: Supplementary file 1 [file vaccines-11-01836-s001.zip › vaccines-2715001-supplementary.pdf]
